# Harmonisation of large-scale, heterogeneous individual participant adverse event data from randomised trials of statin therapy

**DOI:** 10.1177/17407745221105509

**Published:** 2022-07-09

**Authors:** Cholesterol Treatment Trialists’ Collaboration

**Affiliations:** 1 Clinical Trial Service Unit and Epidemiological Studies Unit, Nuffield Department of Population Health, University of Oxford, Oxford, UK; 2 National Health and Medical Research Council, Clinical Trials Centre, University of Sydney, Sydney, NSW, Australia

**Keywords:** Meta-analysis, data harmonisation, standards, randomised trials, Clinical Data Interchange Standards Consortium, Medical Dictionary for Regulatory Activities

## Abstract

**Background:**

Meta-analyses of individual-level data from randomised trials are often required to detect clinically worthwhile effects. The Cholesterol Treatment Trialists’ Collaboration, which includes data from numerous large long-term statin trials, is conducting a review of the effects of statin therapy on all adverse events collected in those trials. This article describes the approaches used and challenges faced to systematically capture and categorise the data.

**Methods:**

Protocols, statistical analysis plans, case report forms, clinical study reports and datasets were obtained, reviewed and checked. Relevant baseline and follow-up data from each trial was then reorganised into standardised formats based upon the Clinical Data Interchange Standards Consortium Study Data Tabulation Model. Adverse event data were organised and coded (automatically or, where necessary, manually) according to a common medical dictionary based upon the Medical Dictionary for Regulatory Activities.

**Results:**

Data from 23 double-blind statin trials and 5 open-label statin trials were provided, either through direct data transfer or through online access platforms. Together, these trials provided 845 datasets containing over 38 million records relating to 30,495 study variables and 181,973 randomised participants. Of the 46 Clinical Data Interchange Standards Consortium Study Data Tabulation Model domains that could potentially have been used to organise the data, the 13 most relevant to the project were identified and utilised, including 6 domains related to post-randomisation adverse events. Nearly 1.2 million adverse events were extracted and mapped to over 45,000 unique adverse event terms. Of these adverse events, 99% were coded to a Medical Dictionary for Regulatory Activities ‘lower level term’, with the remainder coded to a ‘higher level term’ or, very rarely, only a ‘higher level group term’.

**Conclusion:**

In this meta-analysis of adverse event data from the large randomised trials of statins, approaches based on common standards for data organisation and classification have provided a resource capable of allowing reliable and rapid evaluation of any previously unknown benefits or hazards of statin therapy.

## Introduction

The vast majority of interventions have only moderate effects on disease outcomes and hence are impossible to evaluate without careful study. Any clinical study where the main objective is to assess moderate treatment effects must therefore ensure that any biases and any random errors that are inherent in its design are both substantially smaller than the anticipated effect size.^1^ The only way to guarantee the avoidance of moderate biases is to randomise while the only way to guarantee avoidance of moderate random errors is to study large numbers of outcomes. Large-scale randomised controlled trials (RCTs) are therefore typically needed when comparing the effect(s) of any new treatment with a control group. Even then, single randomised controlled trials are rarely large enough to answer all the questions we would like to ask. Consequently, individual participant data meta-analyses of randomised trials are often used to systematically assess the effects of a particular treatment.

The Cholesterol Treatment Trialists’ Collaboration includes large-scale (≥1000 participants), long-term (≥2 years scheduled treatment duration) unconfounded, randomised controlled trials of lipid intervention therapies. To date, reports have focussed on the effects of statin therapy on major vascular events, cause-specific mortality and cancer. In particular, analyses have shown that reduction of low-density lipoprotein cholesterol with a statin reduces the risk of major vascular events without any increase in the risk of non-vascular causes of death or of site-specific cancer in a wide range of people.^2–5^ The results from the Cholesterol Treatment Trialists’ Collaboration have led to significant changes in clinical practice, but in recent years, concerns have been raised that statins may cause a range of other adverse effects (particularly, muscle symptoms). To address these concerns, in 2016, we published a protocol to significantly extend the previous dataset to provide a more complete understanding of the nature and magnitude of the effects of statins.^6^ In particular, all adverse events reported during each trial were requested, in addition to variables that might help in the interpretation of particular events (such as non-trial medications and laboratory results).

Undertaking individual participant data meta-analyses of RCTs can be complex due to the volume of data involved and differences between trials in the way data are collected and organised.^7^ Even seemingly similar RCTs in a single therapeutic area can vary substantially in relation to the type and range of data collected. While some trials make use of widely recognised event-coding systems such as the International Classification of Diseases^8^ or the Medical Dictionary for Regulatory Activities (MedDRA),^9^ versions of such coding systems vary, while other trials may employ country-specific or older dictionary types (such as UK Read codes) or custom codes. In addition, although many investigators remain amenable to the direct transfer of their data to a central coordinating centre, others require the use of data-sharing platforms, which may place restrictions on the software available for analysis and/or the way in which the results of analyses can be extracted.

The purpose of this article is to describe the approaches we used to harmonise the complex, heterogeneous and extensive individual participant data received into a single analysable dataset, the challenges faced in doing so, and the solutions devised to overcome these challenges.

## Methods

**Figure 1.**
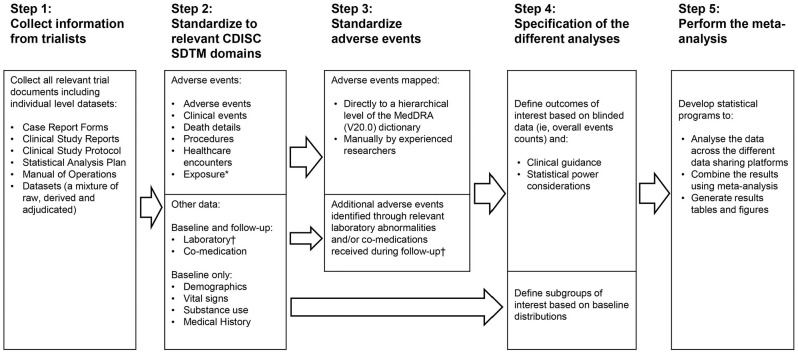
Project flow diagram. CDISC: Clinical Data Interchange Standards Consortium; SDTM: Study Data Tabulation Model. *The exposure domain reported only treatment stops related to adverse events. †The only follow-up laboratory measurements collected were those related to muscle or diabetes outcomes.

The processes through which data (and other associated materials) from each trial were requested and organised (in advance of statistical analyses of the effects of statin therapy on adverse events) are summarised in Figure 1. For each trial, we requested the following: information related to how adverse events were sought, including high-level documentation such as trial protocols; blank copies of the trial case report forms and information on any coding systems used; any available existing tabulations of adverse event data (e.g. in published articles or elsewhere) and all individual-level datasets containing information on adverse events (including the time from randomisation to each adverse event); the timing of and reasons for stopping study treatment; co-medication data and laboratory data or data on physical measurements (e.g. body mass index, blood pressure) or lifestyle characteristics (e.g. smoking). Most trials sent this information directly to the co-ordinating centre in Oxford for storage and processing on secure servers. However, for some trials, this information was made available through the granting of access to a secure online data-sharing platform. These data-sharing platforms, which were only accessible to approved users in the coordinating centre, allowed detailed interrogation and reorganisation of the datasets, but restricted the download of information to summary level results of analyses following submission of a specific data export request. The requirement to use data-sharing platforms for some trials also meant that the planned meta-analyses of the effects of statin therapy on adverse events would necessarily need to be done using a ‘two-stage’ approach.

The project is covered by a UK Research Ethics Committee approval (reference 21/SC/0071).

### Development of a common data format based on Clinical Data Interchange Standards Consortium Study Data Tabulation Model

Existing global standards for data harmonisation were explored for potential use, with the Clinical Data Interchange Standards Consortium Study Data Tabulation Model (CDISC SDTM)^10^ selected as the preferred system due, in part, to its current wide use in RCTs. The CDISC SDTM Implementation Guide describes groups of tabulation datasets (or domains) available for specific data types. All variables within a domain are subdivided into three categories (required, expected and permissible), with these definitions explaining the importance of the variable within the domain. CDISC also allows for ‘customised’ project-specific CDISC SDTM domains or variables to be created.

CDISC SDTM version 3.2 (and version 1.4 of its Implementation Guide) was the version in place at the time of commencement of the project. On review of all available domains in relation to the types of data likely to be received, the study team thought it inappropriate to follow CDISC SDTM rigidly, particularly given that most of the eligible trials included in the project substantially pre-dated CDISC SDTM’s introduction. A pragmatic streamlined approach was therefore adopted, with 13 out of the available 46 domains being considered sufficient for the completion of the CTT IPD meta-analysis project (Table 1). These 13 domains were as follows: Adverse Events, Clinical Events, Co-Medication, Death Details, Demographics, Exposure, Healthcare Encounters, Laboratory Test Results, Medical History, Procedures, Substance Use, Subject Visit and Vital Signs (Full details of the information captured by these 13 domains can be found in Webtable 1 in the Supplemental Material).

**Table 1.** CDISC SDTM (V3.2) domains selected for use in the CTT adverse event project.

SDTM domain	Description
Adverse events	Adverse events which may be reported either as free text or as terms created from a coding system (e.g. MedDRA or International Classification of Diseases).
Clinical events	Trial-specific non-fatal events which were not classified as adverse events.
Co-medication	Concomitant and prior medications/therapies used by the participant. Examples are the concomitant medications/therapies given on an as needed basis and the usual background medications/therapies given for a condition.
Death details	Any fatal events regardless of cause.
Demographics	The parent domain for all other domains including a set of essential standard variables that describe each participant in the clinical study. Examples are the participants’ characteristics at baseline (e.g. treatment allocation, age and race).
Exposure	Reasons for stopping (or adjusting) study treatment. For our purposes, only adverse events resulting in stopping (or adjusting) study treatment were sought.
Healthcare encounters	Inpatient and outpatient healthcare events (e.g. hospitalisations, nursing home stay, rehabilitation facility stays and ambulatory surgery).
Laboratory test results	Laboratory test findings including, but not limited to haematology, clinical chemistry and urinalysis data. For our purposes, only the following laboratory tests were captured: (1) alanine aminotransferase, (2) aspartate aminotransferase, (3) creatine kinase, (4) glucose in blood, (6) high-density lipoprotein cholesterol, (7) low-density lipoprotein cholesterol, (8) triglycerides, (9) total cholesterol, (10) creatinine concentration (serum), (11) estimated glomerular filtration rate, (12) glycosylated haemoglobin and (13) platelets (thrombocytes).
Medical history	The participant’s prior history at the start of the trial (e.g. general medical history, gynaecological history and primary diagnosis).
Procedures	Details describing a participant’s therapeutic and diagnostic procedures (e.g. coronary artery bypass graft, cataract surgery or hip replacement).
Substance use	Substance use information that may be used to assess the efficacy and/or safety of therapies that look to mitigate the effects of chronic substance use. For our purposes, only smoking status and alcohol consumption at baseline were collected.
Subject visit	Information about the timing of participant visits that is otherwise spread over domains that include the visit variables. For our purposes, the subject visit domain was created only when imputation methods were required for timing variables.
Vital signs	Measurements including but not limited to blood pressure, temperature, respiration, body surface area, body mass index, height and weight. For our purposes, only the following measurements were captured: (1) systolic blood pressure, (2) diastolic blood pressure, (3) heart rate, (4) body mass index, (5) height, (6) weight and (7) waist circumference.

CDISC: Clinical Data Interchange Standards Consortium, SDTM: Study Data Tabulation Model; CTT: Cholesterol Treatment Trialists; MedDRA: Medical Dictionary for Regulatory Activities.

A streamlined approach was also adopted to utilisation of variables within each SDTM domain. For example, only 13 types of laboratory test were considered as essential for the analyses and created in the laboratory domain (Table 1). Similarly, for the substance use domain, only baseline smoking status and alcohol consumption were included, while for the medical history domain, only history of cardiovascular diseases and diabetes were utilised.

### Use of a common event-coding dictionary based on MedDRA

Of the 13 SDTM domains considered to be relevant, six (adverse events, clinical events, death details, procedures, healthcare encounters and exposure) pertain to the capture of outcomes that could reasonably be considered to be adverse events when assessing the potential effects of statin therapy. A single categorisation and coding system for adverse events was then needed which would allow for detailed and consistent assessments of any type of adverse event (or group of adverse events) in each trial and, through meta-analyses, across all trials. We selected MedDRA as the reference medical terminology dictionary to harmonise the numerous event-coding formats used in the included trials into a single common language. MedDRA encompasses >77,000 different ‘terms’ based on a five-tiered hierarchical structure ranging from the most granular ‘Lowest Level Term’ to the very general ‘System Organ Class’ (Table 2).^9^

**Table 2.** The five-tiered hierarchical structure of Medical Dictionary for Regulatory Activities (MedDRA).

MedDRA hierarchical level	Description	Total number of available terms
Lower level term	A reflection of how observations might be reported in practice, for example, a verbatim term. Each lower level term is linked to only one preferred term.	77,248
Preferred term	A single descriptor such as a symptom, sign, disease diagnosis, investigation or procedure. Each preferred term has at least one lower level term (itself) as well as synonyms and lexical variants (e.g. abbreviations, different word order).	22,499
Higher level term	A group of preferred terms that are related based on anatomy, pathology, physiology, aetiology or function.	1553
Higher level group term	Related higher level terms are linked to higher level group terms based on anatomy, pathology, physiology, aetiology, or function.	326
System organ class	The final level of the hierarchy where higher level group terms are grouped by aetiology (e.g. infections and infestations), manifestation site (e.g. gastrointestinal disorders) or purpose (e.g. surgical and medical procedures). There is also a system organ class to contain issues pertaining to products and one to contain social circumstances.	27

Adverse event terms (including those given as reasons for stopping study treatment as determined from the SDTM exposure domain) were coded, blind to treatment allocation, to MedDRA version 20.0 (the version in use at the time of commencement of the project). Webtable 2 (Supplemental Material) presents the number of available terms at each of the hierarchical levels in MedDRA version 20.0, organised by system organ class. A computer program was devised to ensure that adverse event terms that directly mapped to an existing MedDRA V20.0 term would be included in analyses not only of that one category, but also of all relevant higher adverse event categories. Initially, a direct match was sought at the lower level term, but if no exact match was found, matches were attempted at higher MedDRA levels (initially at the higher level term, and if that failed then at the higher level group term). For terms that did not directly encode in this way, mapping was done manually by a research team overseen by clinicians. Initially, such terms were checked for obvious errors such as simple typographical errors or changes in word ordering that precluded direct coding. If such basic corrections still failed to yield a direct MedDRA match, a more detailed manual mapping assessment was undertaken, following a coding guidance document developed for the project based upon the MedDRA coding guidance. Such detailed assessment was particularly required when events had been coded as ‘free text’. To ensure consistency, all adverse events were coded by at least two members of the research team, with any discrepancies resolved by discussion. In addition, to ensure medical accuracy, manual mapping of adverse events corresponding to major pre-specified outcomes (such as muscle outcomes and diabetes)^6^ was done by two clinicians. Periodic clinician subsampling of all other encoded adverse events was also undertaken.

### Organisation of trial data prior to analysis

Included trials were categorised into four main groups: double-blind trials of statin therapy versus placebo, open-label trials of statin versus control or usual care, double-blind (i.e. ‘double-dummy’) trials of more intensive versus less intensive statin therapy and open-label trials of more intensive versus less intensive statin therapy.

## Results

### Summary of received data

Data from 28 large-scale clinical trials^11–38^ contributed to this individual participant data meta-analysis. Eight hundred and forty-five datasets were received containing over 38 million records, resulting in 30,495 variables (Table 3). The volume of the data for each trial was driven more by the structure of the questionnaires for each trial and how trialists had structured their own data rather than just the number of participants in the trial. Most trials provided a combination of raw data (i.e. exactly as collected) and derived data (i.e. whereby data cleaning had been applied by the trialists, such as when pre-specified outcomes had been clinically adjudicated). Only two trials provided data in CDISC SDTM format (having applied such formatting retrospectively after completion of the trial).^27,35^

**Table 3.** Characteristics of the 23 double-blind and 5 open-label statin trials.

Study	Year ofpublicationof primaryresults	Number of participants	Treatmentcomparison(mg/day)	Medianfollow-up(years)	Meta-dataCRF (F)CSP (P)CSR (R)	Total number of			Adverse event terms		
						Datasets	Records	Variables	Main medicalterminology^a^	Total	Unique
Statin vs placebo, double-blinded trials											
4S	1994	4444	S20-40 vs placebo	5.4	FP	5	116,502	147	MedDRA*	33,661	1390
WOSCOPS	1995	6595	P40 vs placebo	4.8	FP	23	671,759	509	BMS ICD-9	78,673	7978
CARE	1996	4159	P40 vs placebo	4.9	FPR	9	1,849,516	275	BMS ICD-9	91,635	753
AFCAPS/TexCAPS	1998	6605	L20-40 vs placebo	5.0	P	5	995,156	97	MedDRA*	8143	770
LIPID	1998	9014	P40 vs placebo	5.9	FPR	28	1,347,182	592	ICD-9	57,636	7075
LIPS	2002	1677	F80 vs placebo	4.0	FPR	61	537,144	1145	ICD-10	8867	2392
HPS	2002	20,536	S40 vs placebo	5.2	FP	28	8,152,101	392	MedDRA 14	76,986	2378
PROSPER	2002	5804	P40 vs placebo	3.3	FP	11	628,189	485	Modified ICD-9	131,598	848
ASCOT-LLA	2003	10,240	A10 vs placebo	3.3	FP	10	710,266	466	ICD-9	42,294	1896
ALERT	2003	2102	F40 vs placebo	5.5	FPR	79	1,069,747	5654	ICD-9/MedDRA 5.1	27,114	3589
CARDS	2004	2838	A10 vs placebo	4.2	FP	17	988,712	522	MedDRA 11	26,485	1890
4D	2005	1255	A20 vs placebo	2.7	FP	21	302,442	632	MedDRA 11	6758	461
ASPEN	2006	2410	A10 vs placebo	4.0	FP	18	472,820	379	MedDRA 11	20,323	2861
SPARCL	2006	4731	A80 vs placebo	4.9	FP	14	900,713	512	MedDRA 11	38,811	3539
CORONA	2007	4984	R10 vs placebo	2.8	FPR	62	2,155,519	1716	MedDRA 10	28,899	3450
JUPITER	2008	16,714	R20 vs placebo	1.9	FPR	33	1,540,095	2428	MedDRA 11.1	73,526	5731
GISSI-HF	2008	4574	R10 vs placebo	3.9	F	10	208,489	169	MedDRA 15.1	28,701	537
AURORA	2009	2555	R10 vs placebo	3.9	FPR	42	724,490	1273	MedDRA 11.1	39,512	4013
HOPE-3	2016	12,705	R10 vs placebo	5.6	FP	170	1,415,309	6213	MedDRA 17.1	70,651	7631
Subtotal		123,942				646	24,786,151	23,606		890,273	40,221
Statin versus other control/usual care, open-label trials											
POST-CABG	1997	1351	L40-80 vs L2.5-5	4.3	FP	83	227,740	2821	None	37,164	650
GISSI-P	2000	4271	P20 vs no treatment	1.9	FP	1	4271	795	ICD-9	2777	228
ALLHAT-LLT	2002	10,355	P40 vs no treatment	4.7	FP	10	381,498	103	ICD-9/ICD-10	9932	115
ALLIANCE	2004	2442	A10-80 vs usual care	4.5	FP	15	136,557	344	MedDRA 11	3717	1334
Subtotal		18,419				109	750,066	4063		53,590	2311
More vs less statin, double-blinded trials											
PROVE-IT	2004	4,162	A80 vs P40	2.1	FP	24	603,289	1,161	ICD-9	25,600	1551
A to Z	2004	4,497	S40 then S80vs placebo then S20	2.0	FP	10	386,787	405	MedDRA*	4615	561
TNT	2005	10,001	A80 vs A10	5.0	FP	20	2,676,152	624	MedDRA 11	93,583	3735
SEARCH	2010	12,064	S80 vs S20	7.0	FP	25	7,341,699	344	MedDRA 14	61,379	1303
Subtotal		30,724				79	11,007,927	2534		185,177	6210
More vs less statin, open-label trials											
IDEAL	2005	8888	A40-80 vs S20-40	5	FP	11	1,999,354	292	MedDRA 13	59,129	2281
Subtotal		8888				11	1,999,354	292		59,129	2281
Total		181,973				845	38,543,498	30,495		1,188,169	45,230

S: simvastatin; P: pravastatin; L:lovastatin; F: fluvastatin; A: atorvastatin; R: rosuvastatin; CRF: case report forms; CSP: clinical study protocol; CSR: clinical study report; ICD: International Classification of Diseases; 4S: Scandinavian Simvastatin Survival Study; WOSCOPS: West of Scotland Coronary Prevention Study; CARE: Cholesterol And Recurrent Events; AFCAPS/TexCAPS: Air Force/Texas Coronary Atherosclerosis Prevention Study; LIPID: Long-term Intervention with Pravastatin in Ischaemic Disease; LIPS: Lescol Intervention Prevention Study; HPS: Heart Protection Study; PROSPER: PROspective Study of Pravastatin in the Elderly at Risk; ASCOT-LLA: Anglo-Scandinavian Cardiac Outcomes Trial– Lipid Lowering Arm; ALERT: Assessment of Lescol in Renal Transplantation; CARDS: Collaborative Atorvastatin Diabetes Study; 4D: Die Deutsche Diabetes Dialyse Studie; ASPEN: Atorvastatin Study for Prevention of Coronary Heart Disease Endpoints in Non-Insulin-Dependent Diabetes Mellitus; SPARCL: Stroke Prevention by Aggressive Reduction in Cholesterol Levels; CORONA: Controlled Rosuvastatin Multinational Trial in Heart Failure; JUPITER: Justification for the Use of Statins in Prevention: an Intervention Trial Evaluating Rosuvastatin study group; GISSI-HF: Gruppo Italiano per lo Studio della Sopravvivenza nell’Insufficienza cardiaca; AURORA: A Study to Evaluate the Use of Rosuvastatin in Subjects on Regular Hemodialysis: An Assessment of Survival and Cardiovascular Events; HOPE-3: Heart Outcomes Prevention Evaluation-3 trial; Post-CABG: Post-Coronary Artery Bypass Graft; GISSI-P: Gruppo Italiano per lo Studio della Sopravvivenza nell’Infarto Miocardico; ALLHAT-LLT: Antihypertensive and Lipid-Lowering Treatment to Prevent Heart Attack Trial; ALLIANCE: Aggressive Lipid-Lowering Initiation Abates New Cardiac Events; PROVE-IT: Pravastatin or Atorvastatin Evaluation and Infection Therapy; A to Z: Aggrastat to Zocor; TNT: Treating to New Targets; SEARCH: Study of the Effectiveness of Additional Reductions in Cholesterol and Homocysteine. IDEAL: Incremental Decrease in End Points Through Aggressive Lipid Lowering Study Group.

A small number of participants in the AURORA (n = 218), CORONA (n = 27) and JUPITER (n = 1088) trials withdrew consent for use of their data post-trial, and hence, data from these participants are excluded. The ASCOT-LLA trial excludes 65 participants for whom data were not available due to protocol violations.

a Most trials that used a medical terminology also provided some events in free text format. Some trials used more than one medical terminology.

* MedDRA version was unknown.

### Data harmonisation

The 13 SDTM domains chosen for use in the project yielded a total of 121 variables (of which 13 were additional supplementary variables created specifically for our purposes) (see Webtable 1). However, some trials did not require the creation of all of the 13 selected SDTM domains based on the structure of their data. For example, for trials which reported all of their adverse events (i.e. including fatal and non-fatal study outcomes, hospitalisation and medical procedures) in a single dataset, the creation of the single SDTM adverse event domain was considered sufficient to harmonise such data.

### Mapping of adverse events to MedDRA

The main medical terminology used to encode adverse events in the received trials varied. Seventeen of the 28 trials encoded their data using different versions of MedDRA (Table 3), although in some cases the MedDRA version was not stipulated. All of these 17 trials mapped their adverse event terms to a MedDRA lower level term, although a small subset of events from 3 of the trials were received at the higher level term or higher level group term level (because of a fear by the data providers that provision at the lower level term level could theoretically lead to the discovery of the identity of one or more study participants). Nine trials provided their adverse event data using International Classification of Diseases dictionaries (either version 9 or 10, or a modification or combination of these versions), while one trial used a combination of MedDRA and the International Classification of Diseases. One trial utilised no obviously recognisable coding system, and most trials appeared to use free text (events entered without use of a medical dictionary) on at least some occasions even if a medical coding dictionary was in place. Overall, the 28 trials included a total of approximately 1.2M adverse events corresponding to just over 45,000 unique terms.

Table 4 presents the total number of adverse events mapped directly across the hierarchical levels of MedDRA V20.0, as well as those requiring manual mapping. Of the 1.2M terms, about 780,000 (66%) directly auto-coded to a lower level term (of which nearly 13,000 were unique terms). Less than 1% of events directly auto-coded only at a higher level term or a higher level group term. For those events requiring manual mapping, again the vast majority of terms were coded to a lower level term. The proportion of adverse event terms undergoing auto-encoding versus manual mapping varied considerably between different trials depending on the original coding system used and the structure of the trial case report forms, with auto-encoding ranging from more than 90% to less than 5%.

**Table 4.** Number of total and unique adverse event terms mapped across the hierarchical levels of MedDRA (V20.0).

Type and MedDRA level of coding	Adverse events, N (%)	
	Total^a^	Unique terms^b^
Automatically coded events		
Lower level term	779,449 (65.6%)	11,965 (26.5%)
Higher level term	6216 (0.5%)	659 (1.5%)
Higher level group term	820 (<0.1%)	1 (<0.1%)
Subtotal	786,485 (66.2%)	12,625 (27.9%)
Manually coded events		
Lower level term	391,338 (32.9%)	32,458 (71.8%)
Higher level term	7571 (0.6%)	116 (0.3%)
Higher level group term	2775 (0.2%)	31 (<0.1%)
Subtotal	401,684 (33.8%)	32,605 (72.1%)
Total	1,188,169 (100%)	45,230 (100%)

a Total across all 28 trials. Numbers represent the number of adverse event terms provided by the trialists who were either automatically or manually coded into particular MedDRA levels (lower level, higher level and higher level group).

b As above, but counting the number of unique adverse event terms coded.

Adverse events were not coded to the MedDRA ‘Preferred Term’ level because each preferred term has an identical lower level term available. Thus, once all lower level terms have been coded, coding of preferred terms would provide no additional information.

## Discussion

Our large individual participant data meta-analysis of the effects of statin therapy on all types of adverse event recorded in the large long-term statin trials presented significant data harmonisation challenges, even though all of the trials addressed a similar research question. These challenges largely related to the scale and heterogeneity of the data collected, but also challenges related to a requirement for us to use data-sharing platforms for some trials, as well as other challenges related to the fact that many of the statin trials published their results two or more decades ago.

Over the past two decades, standard methods for harmonising clinical trial data and outcomes have been introduced. Specifically, the CDISC released the first version of SDTM^10^ in 2005 as a standard structure for data tabulation from human clinical trials and non-clinical studies. This is now extensively used by the pharmaceutical industry and academia, and is required by some regulatory authorities such as the United States Food and Drug Administration or Japanese Pharmaceuticals and Medical Devices Agency when receiving data for licensing purposes.

However, there has been a paucity of detailed methodology for harmonising heterogeneous data from numerous different RCTs in individual participant data meta-analyses, especially those pre-dating the CDISC SDTM era (with relatively few trials appearing to have retrospectively fitted historic trial data to such standards). Despite a Preferred Reporting Items for a Systematic Review and Meta-analysis statement for individual participant data meta-analyses being published in 2015 which refers to the need to describe methods of standardising or translating variables within individual participant data datasets to ensure common scales or measurements across studies,^39^ details on how to do this in practice have been lacking. This means that researchers have typically had to develop their own methodology for harmonising numerous datasets in individual participant data meta-analyses by the creation of bespoke systems to convert data from numerous RCTs into a single analysable dataset. This can be challenging and time-consuming.

The system we devised for use in the analysis of adverse events related to statin therapy presents one possible solution to this problem by incorporating methodology based on existing, widely recognised data standards devised by CDISC together with use of the MedDRA dictionary. The strengths of such a system include the transparency of the methodology which may help facilitate the interpretation and generalisability of the results. The ability to create ‘customised’ project-specific CDISC SDTM variables increased the project’s ability to handle very diverse data, while the hierarchical structure of MedDRA allowed mapping of terms at a variety of degrees of term specificity. This was potentially important given the range of terminologies received for reported adverse event terms, although most terms were able to be mapped at the lowest level of MedDRA (i.e. the lower level term) indicating that adverse events captured in our project were relatively granular in their specificity. The fact that terms which did not directly map had clinical oversight of their coding also meant that there was retention of reasonable closeness to original provided terms, which can be challenging if using natural language processing techniques.^40,41^

Limitations of our approach include the fact that despite deployment of streamlined, standards-based methods, the project still required considerable staff resource. Although the CDISC SDTM standard was used to organise and format the data, the corresponding CDISC Analysis Data Model to define datasets and metadata for analysis was not utilised due to analysis programs having been devised for our previous publications (meaning we did not need to explore such CDISC functionality in our project). However, use of CDISC Analysis Data Model methodology may well be worthwhile in future projects that do not have such an established history. Challenges encountered with the MedDRA dictionary included it not affording ready flexibility in terms of creation of project-specific custom codes for terms for which there was no obvious match. Although it is possible to ask MedDRA to add new terms or modify existing terms, these changes take time to approve which can be problematic for ongoing projects that use a specific version of MedDRA. In addition, ‘self-learning’ MedDRA mapping software is not currently available, meaning that such mapping can still be fairly resource intensive. In addition, although it is possible (and entirely reasonable) to adopt a two-stage meta-analysis approach for the assessment of the effects of statins on adverse events, the *requirement* to use data-sharing platforms for some trials does rule out the use of alternative one-stage meta-analysis approaches (which can offer additional flexibility over the two-stage approach).

In summary, through use of systems based upon the widely recognisable standards of CDISC SDTM and MedDRA, we have converted highly heterogeneous legacy data from the large long-term statin trials into one of the richest individual-level datasets in the world, able to address a pressing public health issue. The dataset generated by this project will not only act as a tool to address current concerns regarding statin therapy, but will also act as a resource to test any future hypotheses that may emerge about any other effects of this drug class. The methodological approaches we developed could be easily modified for use in other settings to robustly explore the full effects of widely used interventions.

## Supplemental Material

sj-pdf-1-ctj-10.1177_17407745221105509 – Supplemental material for Harmonisation of large-scale, heterogeneous individual participant adverse event data from randomised trials of statin therapy Supplemental material, sj-pdf-1-ctj-10.1177_17407745221105509 for Harmonisation of large-scale, heterogeneous individual participant adverse event data from randomised trials of statin therapy
