# Supplementary material for: Harmonisation of large-scale, heterogeneous individual participant adverse event data from randomised trials of statin therapy
Source: Clin Trials. 2022 Jul 9;19(6):593–604. doi: 10.1177/17407745221105509 (PMC7613840; doi:10.1177/17407745221105509)
Supplement: sj-pdf-1-ctj-10.1177_17407745221105509 – Supplemental material for Harmonisation of large-scale, heterogeneous individual participant adverse event data from randomised trials of statin therapy [file sj-pdf-1-ctj-10.1177_17407745221105509.pdf]

Online Appendix

Harmonisation of large-scale, heterogeneous individual participant adverse event data from randomised trials of statin therapy

Table of Contents

Webtables **Page**

1. CDISC SDTM (V3.2) domains and variables created for the completion of the CTT adverse event project including appropriate modifications 2

2. Hierarchical levels of MedDRA (V20.0), organised alphabetically by System Organ Class 8

Webtable 1: CDISC SDTM (V3.2) domains and variables created for the completion of the CTT adverse event project including appropriate modifications

Domain model	Variable Name	Variable Label	Type (length)	Description
Adverse Event	STUDYID	Study Identifier	Char ($20)	Unique identifier for a study.
	DOMAIN	Domain Abbreviation	Char ($2)	Two-character abbreviation for the domain.
	USUBJID	Unique Subject Identifier	Char ($50)	Identifier used to uniquely identify a subject across all studies for all applications or submissions involving the product.
	AETERM*	Reported Term for the Adverse Event	Char ($200)	Verbatim name of the event.
	AEDECOD*	Dictionary-Derived Term	Char ($200)	Dictionary-derived text description of AETERM or AEMODIFY. Equivalent to the Preferred Term (PT in MedDRA). The sponsor is expected to provide the dictionary name and version used to map the terms utilizing the define.xml external codelist attributes.
	AESEV	Severity/Intensity	Char ($20)	The severity or intensity of the event. Examples: MILD, MODERATE, SEVERE.
	AESER	Serious Event	Char ($1)	Is this a serious event?
	AEREL	Causality	Char ($200)	Records the investigator's opinion as to the causality of the event to the treatment. ICH E2A and E2B examples include NOT RELATED, UNLIKELY RELATED, POSSIBLY RELATED, RELATED. Controlled Terminology may be defined in the future. Check with regulatory authority for population of this variable.
	AESCONG†	Congenital Anomaly or Birth Defect	Char ($1)	Was the serious event associated with congenital anomaly or birth defect?
	AESDISAB†	Persist or Signif Disability/Incapacity	Char ($1)	Did the serious event result in persistent or significant disability/incapacity?
	AESDTH†	Results in Death	Char ($1)	Did the serious event result in death?
	AESHOSP†	Requires or Prolongs Hospitalization	Char ($1)	Did the serious event require or prolong hospitalization?
	AESLIFE†	Is Life Threatening	Char ($1)	Was the serious event life threatening?
	AESMIE†	Other Medically Important Serious Event	Char ($1)	Do additional categories for seriousness apply?
	AESTDY	Study Day of Start of Selected Adverse Event	Num	Study Day of Start of Selected Adverse Event, measured as integer days.
	SUPP_CTT_AE_TERM	Adverse event term selected for CTT analyses	Char ($200)	Supplementary variable: Adverse event term selected for CTT analyses, either AETERM or AEDECOD.
	SUPP_CTT_AE_STOP	Type of treatment stop‡	Char ($1)	Supplementary variable: Type of treatment stop<br>P = Permanent statin stop<br>T = Temporary statin stop<br>S = Statin stop, unknown if permanent or temporary<br>R = Statin dose reduced<br>N = Did not lead to a statin stop

Clinical Event	STUDYID	Study Identifier	Char ($20)	Unique identifier for a study.
	DOMAIN	Domain Abbreviation	Char ($2)	Two-character abbreviation for the domain.
	USUBJID	Unique Subject Identifier	Char ($50)	Identifier used to uniquely identify a subject across all studies for all applications or submissions involving the product.
	CETERM*	Reported Term for the Clinical Event	Char ($200)	Term for the medical condition or event. Most likely pre-printed on CRF.
	CEDECOD*	Dictionary-Derived Term	Char ($200)	Controlled terminology for the name of the clinical event. The sponsor is expected to provide the dictionary name and version used to map the terms utilizing the define.xml external codelist attributes
	CESEV	Severity/Intensity	Char ($50)	The severity or intensity of the event. Examples: MILD, MODERATE, SEVERE
	CEDY	Study Day of Event Collection	Num	Study day of clinical event collection, measured as integer days.
	SUPP_CTT_CE_TERM	Clinical event term selected for CTT analyses	Char ($200)	Supplementary variable: Clinical event term selected for CTT analyses, either CETERM or CEDECOD.
Death Details	STUDYID	Study Identifier	Char ($20)	Unique identifier for a study.
	DOMAIN	Domain Abbreviation	Char ($2)	Two-character abbreviation for the domain.
	USUBJID	Unique Subject Identifier	Char ($50)	Identifier used to uniquely identify a subject across all studies for all applications or submissions involving the product.
	DDORRES	Result or Finding as Collected	Char ($200)	Result of the test defined in DDTEST, as originally received or collected.
	DDDY	Study Day of Collection	Num	Study day of the collection, in integer days.
Health encounters	STUDYID	Study Identifier	Char ($20)	Unique identifier for a study.
	DOMAIN	Domain Abbreviation	Char ($2)	Two-character abbreviation for the domain.
	USUBJID	Unique Subject Identifier	Char ($50)	Identifier used to uniquely identify a subject across all studies for all applications or submissions involving the product.
	HOTERM*	Reported Term for the Healthcare Encounter	Char ($200)	Verbatim or preprinted CRF term for the healthcare encounter.
	HODECOD*	Dictionary-Derived Term for the Healthcare Encounter	Char ($200)	Dictionary or sponsor-defined derived text description of HOTERM or the modified topic variable (HOMODIFY).
	HOSTDY	Study Day of Start of Healthcare Encounter	Num	Study Day of Start of Selected Healthcare Encounter, measured as integer days.
	SUPP_CTT_HO_TERM	Hospitalisation event term selected for CTT analyses	Char ($200)	Supplementary variable: Hospitalisation event term selected for CTT analyses, either HOTERM or HODECOD.
Procedures	STUDYID	Study Identifier	Char ($20)	Unique identifier for a study.
	DOMAIN	Domain Abbreviation	Char ($2)	Two-character abbreviation for the domain.
	USUBJID	Unique Subject Identifier	Char ($50)	Identifier used to uniquely identify a subject across all studies for all applications or submissions involving the product.
	PRTRT*	Reported Name of Procedure	Char ($200)	Name of procedure performed, either pre-printed or collected on a CRF.
	PRDECOD*	Standardized Procedure Name	Char ($200)	Standardized or dictionary-derived name of PRTRT. The sponsor is expected to provide the dictionary name and version used to map the terms in the define.xml external codelist attributes. If an intervention term does not have a decode value in the dictionary then PRDECOD will be null.

	PRSTDY	Study Day of Start of Procedure	Num	Study day of start of procedure, measured as integer days.
	SUPP_CTT_PR_TERM	Procedure event term selected for CTT analyses	Char ($200)	Supplementary variable: Procedure event term selected for CTT analyses, either PRTRT or PRDECOD.
Concomitant/Prior Medications	STUDYID	Study Identifier	Char ($20)	Unique identifier for a study.
	DOMAIN	Domain Abbreviation	Char ($2)	Two-character abbreviation for the domain.
	USUBJID	Unique Subject Identifier	Char ($50)	Identifier used to uniquely identify a subject across all studies for all applications or submissions involving the product.
	CMTRT*	Reported Name of Drug, Med, or Therapy	Char ($200)	Verbatim medication name that is either pre-printed or collected on a CRF.
	CMDECOD*	Standardized Medication Name	Char ($200)	Standardized or dictionary-derived text description of CMTRT or CMMODIFY. Equivalent to the generic medication name in WHO Drug. The sponsor is expected to provide the dictionary name and version used to map the terms utilizing the define.xml external codelist attributes. If an intervention term does not have a decode value in the dictionary then CMDECOD will be left blank.
	CMENDY	Study Day of End of Medication	Num	Study day of end of medication relative to the sponsor-defined RFSTDTC.
	CMSTDY	Study Day of Start of Selected Co-medication term	Num	Study Day of Start of Medication, measured as integer days.
	SUPP_CTT_CM_TERM	Co-medication term selected for CTT analyses	Char ($200)	Supplementary variable: Co-medication term selected for CTT analyses either CMTRT or CMDECOD.
	SUPP_CMBLFL	Baseline Flag	Char ($1)	Supplementary variable: Indicator used to identify a baseline value. The value should be “Y” or null.
Demographics	STUDYID	Study Identifier	Char ($20)	Unique identifier for a study.
	DOMAIN	Domain Abbreviation	Char ($2)	Two-character abbreviation for the domain.
	USUBJID	Unique Subject Identifier	Char ($50)	Identifier used to uniquely identify a subject across all studies for all applications or submissions involving the product. This must be a unique number, and could be a compound identifier formed by concatenating STUDYID-SITEID-SUBJID.
	AGE	Age	Num	Age expressed in AGEU. May be derived from RFSTDTC and BRTHDTC, but BRTHDTC may not be available in all cases (due to subject privacy concerns).
	AGEU	Age Units	Char ($20)	Units associated with AGE.
	SEX	Sex	Char ($1)	Sex of the subject.
	RACE*	Race	Char ($20)	Race of the subject. Sponsors should refer to “Collection of Race and Ethnicity Data in Clinical Trials” (FDA, September 2005) for guidance regarding the collection of race (http://www.fda.gov/RegulatoryInformation/Guidances/ucm126340.htm) See Assumption below regarding RACE.
	ETHNIC*	Ethnicity	Char ($20)	The ethnicity of the subject. Sponsors should refer to “Collection of Race and Ethnicity Data in Clinical Trials” (FDA, September 2005) for guidance regarding the collection of ethnicity (http://www.fda.gov/RegulatoryInformation/Guidances/ucm126340.htm).
	ACTARMCD	Actual Arm Code	Char ($200)	Code of actual Arm. When an Arm is not planned (not in Trial Arms), ACTARMCD will be UNPLAN. Randomized subjects who were not treated will be given a value of NOTTRT. Values should be “SCRNFAIL” for screen failures and “NOTASSGN” for subjects not assigned to treatment. Restricted to values in Trial Arms in all other cases. ACTARMCD is limited to 20 characters and does not have special character restrictions. The maximum length of ACTARMCD is longer than for other short variables to accommodate the kind of values that are likely to be needed for crossover trials.
	ACTARM	Description of Actual Arm	Char ($200)	Description of actual Arm. When an Arm is not planned (not in Trial Arms), ACTARM will be “Unplanned Treatment”. Randomized subjects who were not treated will be given a value of “Not Treated”. Values should be “Screen Failure” for screen failures and “Not Assigned” for subjects not assigned to treatment. Restricted to values in Trial Arms in all other cases.

	SUPP_CTT_END_DAY	Reference End Day for the subject after randomization	Num	Supplementary variable: Reference End Day for the subject after randomization, measured as integer days. Defined as date of death or last date patient known to be alive.
	SUPP_AGEGRP	Age in 5 years categories	Char ($20)	Supplementary variable: Age in 5 years categories. Required only if variable AGE was missing.
Exposure	STUDYID	Study Identifier	Char ($20)	Unique identifier for a study.
	DOMAIN	Domain Abbreviation	Char ($2)	Two-character abbreviation for the domain.
	USUBJID	Unique Subject Identifier	Char ($50)	Identifier used to uniquely identify a subject across all studies for all applications or submissions involving the product.
	EXADJ	Reason for Dose Adjustment	Char ($200)	Describes reason or explanation of why a dose is adjusted.
	EXENDY	Study Day of End of Treatment	Num	Study day of end of treatment.
	SUPP_CTT_EX_STOP	Type of treatment stop‡	Char ($1)	Supplementary variable: Type of treatment stop<br>P = Permanent statin stop<br>T = Temporary statin stop<br>S = Statin stop, unknown if permanent or temporary<br>R = Statin dose reduced<br>N = Did not lead to a statin stop
Laboratory Test Results	STUDYID	Study Identifier	Char ($20)	Unique identifier for a study.
	DOMAIN	Domain Abbreviation	Char ($2)	Two-character abbreviation for the domain.
	USUBJID	Unique Subject Identifier	Char ($50)	Identifier used to uniquely identify a subject across all studies for all applications or submissions involving the product.
	LBTESTCD	Lab Test or Examination Short Name	Char ($200)	Short name of the measurement, test, or examination described in LBTEST. It can be used as a column name when converting a dataset from a vertical to a horizontal format. The value in LBTESTCD cannot be longer than 8 characters, nor can it start with a number (e.g.”1TEST”). LBTESTCD cannot contain characters other than letters, numbers, or underscores. Examples: ALT, LDH.
	LBTEST	Lab Test or Examination Name	Char ($200)	Verbatim name of the test or examination used to obtain the measurement or finding. Note any test normally performed by a clinical laboratory is considered a lab test. The value in LBTEST cannot be longer than 40 characters. Examples: Alanine Aminotransferase, Lactate Dehydrogenase.
	LBORRES	Result or Finding in Original Units	Char ($20)	Result of the measurement or finding as originally received or collected.
	LBORRESU	Original Units	Char ($20)	Original units in which the data were collected. The unit for LBORRES. Example: g/L.
	LBORNRHI	Reference Range Upper Limit in Orig Unit	Char ($20)	Upper end of reference range for continuous measurements in original units. Should be populated only for continuous results.
	LBSTRESN	Numeric Result/Finding in Standard Units	Num	Used for continuous or numeric results or findings in standard format; copied in numeric format from LBSTRESC. LBSTRESN should store all numeric test results or findings.
	LBSTRESU	Standard Units	Char ($20)	Standardized unit used for LBSTRESC or LBSTRESN.
	LBSTNRHI	Reference Range Upper Limit-Std Units	Num	Upper end of reference range for continuous measurements in standardized units. Should be populated only for continuous results.
	LBNRIND	Reference Range Indicator	Char ($20)	1. Indicates where the value falls with respect to reference range defined by LBORNRLO and LBORNRHI, LBSTNRLO and LBSTNRHI, or by LBSTNRC. Examples: NORMAL, ABNORMAL, HIGH, LOW. 2. Sponsors should specify in the study metadata (Comments column in the define.xml) whether LBNRIND refers to the original or standard reference ranges and results. 3.Should not be used to indicate clinical significance.
	LBBLFL	Baseline Flag	Char ($1)	Indicator used to identify a baseline value. The value should be “Y” or null.

	LBFAST	Fasting Status	Char ($1)	Indicator used to identify fasting status such as Y, N, U, or null if not relevant. For CTT purposes, it is only required for glucose tests.
	LBDRVFL	Derived Flag	Char ($1)	Used to indicate a derived record. The value should be Y or null. Records that represent the average of other records, or do not come from the CRF, or are not as originally received or collected are examples of records that might be derived for the submission datasets. If LBDRVFL=Y, then LBORRES may be null, with LBSTRESC, and (if numeric) LBSTRESN having the derived value.
	LBDY	Study Day of Specimen Collection	Num	Study day of specimen collection, measured as integer days.
	LBTPT	Planned Time Point Name	Char ($200)	Planned Time Point Name Required only for Oral Glucose Tolerance Tests (OGTTs) measurements
Medical History	STUDYID	Study Identifier	Char ($20)	Unique identifier for a study.
	DOMAIN	Domain Abbreviation	Char ($2)	Two-character abbreviation for the domain.
	USUBJID	Unique Subject Identifier	Char ($50)	Identifier used to uniquely identify a subject across all studies for all applications or submissions involving the product.
	MHTERM*	Reported Term for the Medical History	Char ($200)	Verbatim or preprinted CRF term for the medical condition or event.
	MHDECOD*	Dictionary-Derived Term	Char ($200)	Dictionary-derived text description of MHTERM or MHMODIFY. Equivalent to the Preferred Term (PT in MedDRA). The sponsor is expected to provide the dictionary name and version used to map the terms utilizing the define.xml external codelist attributes.
	MHCAT	Category for Medical History	Char ($200)	Used to define a category of related records. Examples: CARDIAC or GENERAL
	MHDY	Study Day of History Collection	Num	Study day of medical history collection, measured as integer days.
	SUPP_CTT_MH_TERM	Medical history term selected for CTT analyses	Char ($200)	Supplementary variable: Medical history term selected for analyses
Substance Use	STUDYID	Study Identifier	Char ($20)	Unique identifier for a study.
	DOMAIN	Domain Abbreviation	Char ($2)	Two-character abbreviation for the domain.
	USUBJID	Unique Subject Identifier	Char ($50)	Identifier used to uniquely identify a subject across all studies for all applications or submissions involving the product.
	SUTRT	Reported Name of Substance	Char ($200)	Substance name. Examples: Cigarettes, Coffee.
	SUDOSTXT	Substance Use Consumption Text	Char ($200)	Substance use consumption amounts or a range of consumption information collected in text form. Not populated if SUDOSE is populated.
	SUSTDY	Study Day of Start of Substance Use	Num	Study day of start of substance use relative to randomization date, measured as integer days.
	SUPP_SUBLFL	Baseline flag	Char ($1)	Supplementary variable: Baseline flag
Subject Visit	STUDYID	Study Identifier	Char ($20)	Unique identifier for a study.
	DOMAIN	Domain Abbreviation	Char ($2)	Two-character abbreviation for the domain.
	USUBJID	Unique Subject Identifier	Char ($50)	Identifier used to uniquely identify a subject across all studies for all applications or submissions involving the product.
	VISITNUM	Visit Number	Num	1. Clinical encounter number. (Decimal numbering may be useful for inserting unplanned visits.) 2. Numeric version of VISIT, used for sorting.
	VISIT	Visit Name	Char ($50)	1. Protocol-defined description of clinical encounter. 2. May be used in addition to VISITNUM and/or VISITDY as a text description of the clinical encounter.

	SVSTDY	Study Day of Start of Visit	Num	Study day of start of visit relative to randomization date, measured as integer days.
Vital Signs	STUDYID	Study Identifier	Char ($20)	Unique identifier for a study.
	DOMAIN	Domain Abbreviation	Char ($2)	Two-character abbreviation for the domain.
	USUBJID	Unique Subject Identifier	Char ($50)	Identifier used to uniquely identify a subject across all studies for all applications or submissions involving the product.
	VSTESTCD	Vital Signs Test Short Name	Char ($200)	Short name of the measurement, test, or examination described in VSTEST. It can be used as a column name when converting a dataset from a vertical to a horizontal format. The value in VSTESTCD cannot be longer than 8 characters, nor can it start with a number (e.g.”1TEST”). VSTESTCD cannot contain characters other than letters, numbers, or underscores. Examples: SYSBP, DIABP, BMI.
	VSTEST	Vital Signs Test Name	Char ($200)	Verbatim name of the test or examination used to obtain the measurement or finding. The value in VSTEST cannot be longer than 40 characters. Examples: Systolic Blood Pressure, Diastolic Blood Pressure, Body Mass Index.
	VSORRES	Result or Finding in Original Units	Char ($20)	Result of the vital signs measurement as originally received or collected.
	VSORRESU	Original Units	Char ($20)	Original units in which the data were collected. The unit for VSORRES. Examples: IN, LB, BEATS/MIN.
	VSSTRESN	Numeric Result/Finding in Standard Units	Num	Used for continuous or numeric results or findings in standard format; copied in numeric format from VSSTRESC. VSSTRESN should store all numeric test results or findings.
	VSSTRESU	Standard Units	Char ($20)	Standardized unit used for VSSTRESC and VSSTRESN.
	VSBLFL	Baseline Flag	Char ($1)	Indicator used to identify a baseline value. The value should be “Y” or null.
	VSDY	Study Day of Vital Signs	Num	Study day of vital signs measurements, measured as integer days.
	SUPP_VSORRESGRP	Results as originally received in categories	Char ($20)	Supplementary variable: Result of the vital signs measurement as originally received or collected (ie, categorized). Required only if variable VSORRES was missing

* At least one of the two variables within each domain was required for the completion of this project † Only to be created if variable AESER cannot be created.

Webtable 2: Hierarchical levels of MedDRA (V20.0), organised alphabetically by System Organ Class

	Number of terms at each sublevel			
**System Organ Class**	**Higher Level Group Term**	**Higher Level Term**	**Preferred Term***	**Lower Level Term**
Blood and lymphatic system disorders	10	32	284	1135
Cardiac disorders	9	29	335	1430
Congenital, familial and genetic disorders	19	98	1311	3462
Ear and labyrinth disorders	5	14	87	428
Endocrine disorders	7	25	187	668
Eye disorders	12	50	592	2432
Gastrointestinal disorders	21	88	847	3807
General disorders and administration site conditions	7	36	977	2412
Hepatobiliary disorders	3	14	191	650
Immune system disorders	4	16	142	457
Infections and infestations	12	149	1907	7079
Injury, poisoning and procedural complications	8	71	1128	6496
Investigations	23	106	5518	13512
Metabolism and nutrition disorders	13	46	277	939
Musculoskeletal and connective tissue disorders	10	38	444	2511
Neoplasms benign, malignant and unspecified (including cysts and polyps)	39	194	1942	8490
Nervous system disorders	18	76	938	3531
Pregnancy, puerperium and perinatal conditions	8	32	222	1633
Product issues	2	21	145	589
Psychiatric disorders	23	69	508	2341
Renal and urinary disorders	8	28	350	1202
Reproductive system and breast disorders	14	44	475	1715
Respiratory, thoracic and mediastinal disorders	10	33	519	1696
Skin and subcutaneous tissue disorders	8	47	489	2054
Social circumstances	7	20	271	635
Surgical and medical procedures	19	141	2107	4632
Vascular disorders	11	36	306	1312
**Total number of terms**	**326**	**1553**	**22499**	**77248**

Lower level terms are mapped only to their *primary* SOC. *Adverse events were not coded to the MedDRA 'Preferred Term' (PT) level because each PT has an identical lower level term (LLT) available.
